# Modulation of Untruthful Responses with Non-Invasive Brain Stimulation

**DOI:** 10.3389/fpsyt.2012.00097

**Published:** 2013-02-26

**Authors:** Shirley Fecteau, Paulo Boggio, Felipe Fregni, Alvaro Pascual-Leone

**Affiliations:** ^1^Berenson-Allen Center for Non-invasive Brain Stimulation, Harvard Medical School and Beth Israel Deaconess Medical CenterBoston, MA, USA; ^2^Laboratory of Canada Research Chair in Cognitive Neuroplasticity, Centre Interdisciplinaire de recherche en réadaptation et intégration sociale, Centre de Recherche de l’Institut Universitaire en Santé Mentale de Québec, Medical School, Laval UniversityQuebec City, Canada; ^3^Núcleo de Neurociências, Centro de Ciências Biológicas e da Saúde, Universidade Presbiteriana MackenzieSao Paulo, Brazil; ^4^Laboratory of Neuromodulation, Spaulding Rehabilitation Hospital, Harvard Medical SchoolBoston, USA; ^5^Institut Guttmann, Universitat Autonoma BarcelonaBarcelona, Spain

**Keywords:** deception, dorsolateral prefrontal cortex, brain stimulation, verbal communication

## Abstract

Deceptive abilities have long been studied in relation to personality traits. More recently, studies explored the neural substrates associated with deceptive skills suggesting a critical role of the prefrontal cortex. Here we investigated whether non-invasive brain stimulation over the dorsolateral prefrontal cortex (DLPFC) could modulate generation of untruthful responses about subject’s personal life across contexts (i.e., deceiving on guilt-free questions on daily activities; generating previously memorized lies about past experience; and producing spontaneous lies about past experience), as well as across modality responses (verbal and motor responses). Results reveal that real, but not sham, transcranial direct current stimulation (tDCS) over the DLPFC can reduce response latency for untruthful over truthful answers across contexts and modality responses. Also, contexts of lies seem to incur a different hemispheric laterality. These findings add up to previous studies demonstrating that it is possible to modulate some processes involved in generation of untruthful answers by applying non-invasive brain stimulation over the DLPFC and extend these findings by showing a differential hemispheric contribution of DLPFCs according to contexts.

## Introduction

Deception is generally defined as deliberately intending to mislead another person by falsification of truthful information (Vrij, 2001; DePaulo et al., 2003; Spence et al., 2004). Several types of deception exist, but they all seem to share a complex neural network with the prefrontal cortex as putative conductor (e.g., Spence et al., 2004; Gombos, 2006). Deceptive abilities appear early in ontogenesis and parallel the developmental course of intricate complex social and communication behaviors along with maturity of executive functions, especially inhibitory control. Although humans are experts at deceiving (lying seems to be a daily life event: DePaulo et al., 2003), it generally requires additional cognitive processing than being truthful (Spence et al., 2004; Gombos, 2006; Vrij et al., 2006; but see DePaulo et al., 2003). The more complex a lie is, the greater the cognitive load (Vrij and Mann, 2001). Various behavioral cues have been identified and suggested to be a signature of this increased cognitive burden. For instance, verbal (e.g., increased pauses; Mann et al., 2002; DePaulo et al., 2003; Vrij, 2005), vocal (e.g., higher pitch; DePaulo et al., 2003), and non-verbal cues (e.g., reduced bodily movements, increased gaze aversion; Vrij and Mann, 2001; Mann et al., 2002; DePaulo et al., 2003; Nunez et al., 2005) have been noted during false statements. However, reliability of these cues to discriminate deceptive from truthful responses remain very poor (Vrij, 2001; DePaulo et al., 2003; Masip et al., 2003; Vrij et al., 2006, 2007, 2008).

One indicator of deceit that has shown consistency in experimental setting is latency of response time. It takes longer to provide untruthful than truthful answers (Spence et al., 2001, 2004; Farrow et al., 2003; Walczyk et al., 2003; Johnson et al., 2004, 2007; Nunez et al., 2005). Moreover, it is difficult to alter response latency by strategic manipulation, like other cues such as gaze aversion or body gesture. Despite being informed on how to modulate their response time, subjects failed at mitigating this response time effect (Seymour et al., 2000). Even the level of stake (Vrij et al., 2008), motivation, and transgression (DePaulo et al., 2003) do not appear to influence this lengthened response time (but see Verschuere et al., 2009).

The objective of this work was to investigate whether this lengthening in response latency associated with untruthful answers can be modulated using transcranial direct current stimulation (tDCS) over the dorsolateral prefrontal cortex (DLPFC) across contexts and modality responses. The overreaching neurobiological conceptualization here is the idea that deceptive behaviors regardless of contexts can be learned and trained involving the DLPFC. A better understanding of the role of the DLPFC in deception is important because it may also shed light on impaired neurobehavioral substrates in populations who are disabled with compulsive deception (e.g., antisocial personality disorder).

We conducted a series of experiments with healthy volunteers to assess the effects of tDCS over the DLPFC in three different contexts: (1) generating untruthful answers about daily personal information that does not elicit significant guilt (Task 1), (2) generating a coherent lie that was previously memorized, and (3) producing spontaneously a coherent lie (Task 2). Control experiments included a task on the ability to generate spontaneous verbal responses (Task 3) and the Stroop interference (Task 4). Personality profiles were characterized with the Psychopathic Personality Inventory (PPI).

## Materials and Methods

Thirty-six subjects (11 men; three left-handed; mean age of 21.6 ± 3.8 years) took part in the study, which comprised three experimental tasks and two control tasks. Although studies have reported bilateral prefrontal activations, including in DLPFCs, associated with deceptive answers with a right dominance and have been correlated with response latency (e.g., Gamer et al., 2007), the hemispheric contribution is not clear yet (e.g., Spence et al., 2004). We therefore included three types of electrode arrangements. One arrangement was with the anodal electrode placed over the right DLPFC coupled with the cathodal electrode over the contralateral DLPFC (referred here as ‘right anodal/left cathodal’), which is known to enhance excitability in the right DLPFC and decrease it in the left DLPFC. A second stimulation condition was with the anodal electrode placed over the left DLPFC coupled with the cathodal electrode over the contralateral DLPFC (referred as ‘left anodal/right cathodal’) and is known to activate the left and suppress the right DLPFC excitability. The third condition was a sham stimulation control with both electrodes placed over the DLPFC (half of the subjects with the sham left anodal/right cathodal arrangement, the other half with the sham right anodal/left cathodal arrangement). Subjects were pseudo-randomly assigned to receive either right anodal/left cathodal (*N* = 12; five men; one left-handed; mean age of 22.2 ± 3.3 years), left anodal/right cathodal (*N* = 12; 2 men; one left-handed; mean age of 20.3 ± 1.9 years), sham stimulation (*N* = 12; four men; one left-handed; mean age of 22.4 ± 5.4 years). All participants were college students. They were not taking medications, had no history of neurological or psychiatric disorders and had normal physical and neurological exams. They were screened for contraindications for non-invasive brain stimulation. All were naive to brain stimulation and were not informed about the main experimental variables tested (i.e., response latencies). They gave informed written consent prior to entering the study, which was approved by the local ethics committee. The study was performed at Mackenzie University (Sao Paulo, Brazil).

**Figure 1:**
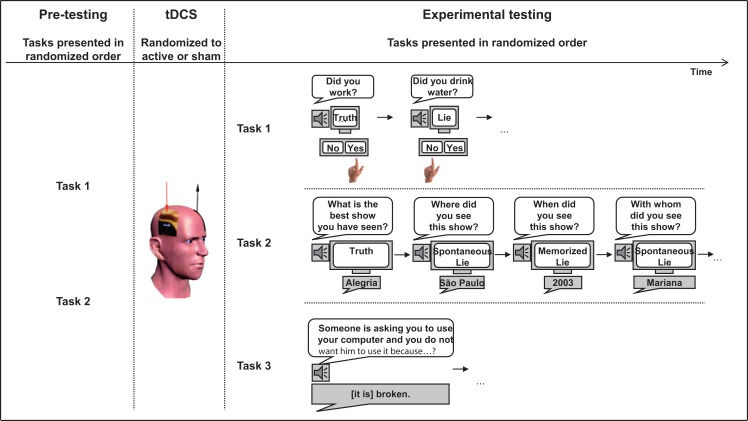
**Design of the study**. Participants first performed Tasks 1 and 2 (prior stimulation), they then received active or sham tDCS, and they finally performed Tasks 1 and 2 again, as well as the Task 3.

Each participant performed a total of four tasks (see Figure 1). The order of tasks was counterbalanced across subjects. Pre-testing was done before stimulation and then all tasks were tested one after the other after stimulation. The four tasks were completed within 30 min after the end of stimulation. The four tasks, as well as the PPI, were administrated by investigators blinded to stimulation condition and experimental variables tested.

### Experimental Task 1: Generating untruthful responses about personal daily activities

The goal of Task 1 was to test whether tDCS over the DLPFC could modulate untruthful answers in the context of personal daily activities. This task was based on Spence et al. (2001). Before brain stimulation (the same day), participants filled out a form that included 33 questions about their personal daily activities (Instructions: “In the course of today, have you done any of the following?”; see Figure 1). This form provided us with *truthful* answers about each participant’s daily activities. After receiving brain stimulation, participants were asked to answer (*yes*/*no*) on their daily activities according to the cue provided on a computer screen (*truth*/*lie*). The questions were the same as those asked before stimulation. Each question was asked twice, half presented with the cue *truth*, the other half with the cue *lie*. Questions were auditorily presented and subjects had to give a motor response using the computer keyboard. Half of the subjects had to press the key “v” for *yes* and the key “b” for *no*, the other half had to press the opposite key setting: “b” for *yes* and “v” for *no*. The order of the questions and the cues was pseudo-randomized. Level of guilt to lie about these activities was judged from an independent group of healthy volunteers (*N* = 5; two men; mean age 28.3 ± 2.5 years) on visual analog scales (with “0” defined as *not at all* and “100” as *very much*). Average rating of level of guilt of all questions was 17.6% (SD = 23.3%).

### Experiment Task 2: Deceiving about personal past experience

The aim of Task 2 was to test whether tDCS over the DLPFC could change untruthful responses about personal past experience with either memorized lies or spontaneous lies. This experiment was based on work from Ganis et al. (2003). Immediately before stimulation, subjects were asked to fill out a questionnaire (see Figure 1). The questions were: (1) what is the best movie you have seen, where, when and with whom did you see it, and (2) what is the best show you have seen, where, when, and with whom did you see it. They were then asked to make up plausible lies to these same questions and to memorize them because they would be asked after stimulation to retrieve these memorized lies. After stimulation, they had to answer the same questions (e.g., what is the best show you have seen?) three times according to the cue provided (*truth*, *memorized lie*, and *spontaneous lie*). The questions were auditorily presented for maximizing ecological validity and subjects had to provide a verbal answer.

### Control Task 3: Generation of spontaneous verbal responses

In this control task, we tested for possible effects of tDCS over DLPFC on the ability to spontaneously generate verbal responses as it has been shown that TMS over DLPFC can reduce response time on verbal fluency tasks (Iyer et al., 2005). Participants had to provide a viable response to nine open-ended questions. The instructions were “Here are different scenarios. You have to come up with a plausible answer for each scenario. Try to be as convincing as possible. Your answers will be recorded and there will be individuals, who do not know that all of your answers are all made up answers, who will try to identify which of your answers are truthful. Here is an example: Someone is asking you to use your computer and you do not want him to use it because…”. The scenarios were audio-recorded and subjects had to provide a verbal answer thus maximizing ecological validity as in Task 2. The order of the scenarios was pseudo-randomized.

### Control Task 4: Stroop interference

We tested for possible effects of brain stimulation on inhibitory control functions using the Stroop task. Stimulation over the DLPFC likely modulates activity also in neighboring regions such as the orbitofrontal area, which is involved in inhibitory control functions (Elliot and Deakin, 2005). Modulation of inhibitory functions could impact deceptive skills that would not be specific to the ability of being deceptive. Participants were therefore asked to perform the Stroop task, a standardized paradigm to measure non-specific inhibitory control related to prefrontal cortex, before and after stimulation. We measured the Stroop interference, which is characterized by slower response in naming incongruent words (i.e., the word *red* printed in green ink) as compared to color congruent words (Stroop, 1935).

### Psychopathic personality inventory

Personality profile of participants was assessed because personality traits may contribute to the ability of deceiving. To test for possible differences in personality features between groups, participants filled out the PPI (Lillienfeld and Andrews, 1996) before the stimulation session. The PPI comprises eight subscales: Machiavellian egocentricity, social potency, fearlessness, coldheartedness, impulsive non-conformity, blame externalization, carefree non-planfulness, and stress immunity.

### Transcranial direct current stimulation

Direct current was induced by two saline-soaked surface sponge electrodes (35 cm^2^) and delivered in a double-blinded fashion by a battery-driven, constant current stimulator. The device used, developed by our group, is particularly reliable for double-blind studies: a switch can be activated to interrupt the electrical current while maintaining the *ON* display and showing the stimulation parameters throughout the procedure to the experimenter and participant. For right anodal/left cathodal stimulation, the anode electrode was placed over right F4 (international EEG 10/20 system) and the cathode electrode over left F3. For left anodal/right cathodal stimulation, the polarity was reversed: the anode electrode was placed over F3 (EEG 10/20 system) and the cathode electrode over F4. For active stimulation, participants received a constant current of 2 mA intensity. Stimulation was delivered for 20 min and participants performed the tasks immediately after the end of the stimulation session. For sham stimulation, the electrodes were placed at the same position as for active stimulation (F3 and F4), but the stimulator was turned on only for the first 30 s so participants felt the initial itching sensation associated with the stimulation, but received no active current for the rest of the stimulation period. This method of sham stimulation has been shown to be reliable (Gandiga et al., 2006).

### Data analysis

Tasks 1, 2, and 3 were administrated using PsyScope software X B41 running on a PowerBook G4 (Apple Inc., Cuppertino, CA, USA).

In Task 1 (Generating untruthful responses about personal daily activities), the outcome measure was the difference in motor response latency in milliseconds between the lie and truth conditions. Only response latencies of *correct* answers at both lie and truth conditions were analyzed based on participants’ information on their daily activities collected before brain stimulation. Response latencies were then averaged for each subject and across stimulation conditions. Due to technical problems during Task 1, data from three subjects were not recorded (one in the right anodal/left cathodal, one in the left anodal/right cathodal, and one in the sham group).

In Task 2 (Generating untruthful responses about personal past experience) and in Task 3 (Generation of spontaneous verbal responses), the outcome measure was the verbal response latency, i.e., time elapse between the end of the verbal question and the onset of subjects’ correct (memorized lies) or coherent, plausible answer (spontaneous lies and spontaneous verbal responses). Only one response was excluded because it was not plausible: “Bob Marley” (Task 2). Vocal answers were recorded using Voice Editing Premium Edition recorder (Panasonic Corporation). Response latency was calculated as the time between the end of the instruction and the onset of the first word produced by the subject. Filler words such as “my friend…” were discarded. Response latency was measured in milliseconds using praat (http://www.praat.org) and then averaged across conditions: truth, memorized lies, spontaneous lies, and spontaneous verbal responses.

For all experiments, response latencies were measured by two individuals blinded to stimulation condition. Outliers, defined as 2 SD above or below individual mean of onset of response latency for each condition and participant, were excluded. Analyses were performed using SAS (SAS Institute Inc, NC, USA). Results with a *p*-value of ≤0.05 were considered significant for all statistical analyses.

## Results

None of the volunteers reported adverse effects during or after brain stimulation. Most participants perceived a slight itching sensation under the electrodes during the first seconds of stimulation. When explicitly asked at the end of the study whether they believe having received active or sham stimulation, all participants believed to have undergone active stimulation, suggesting successful blinding of the sham stimulation condition.

### Experimental Task 1: Generating untruthful responses about personal daily activities

**Figure 2:**
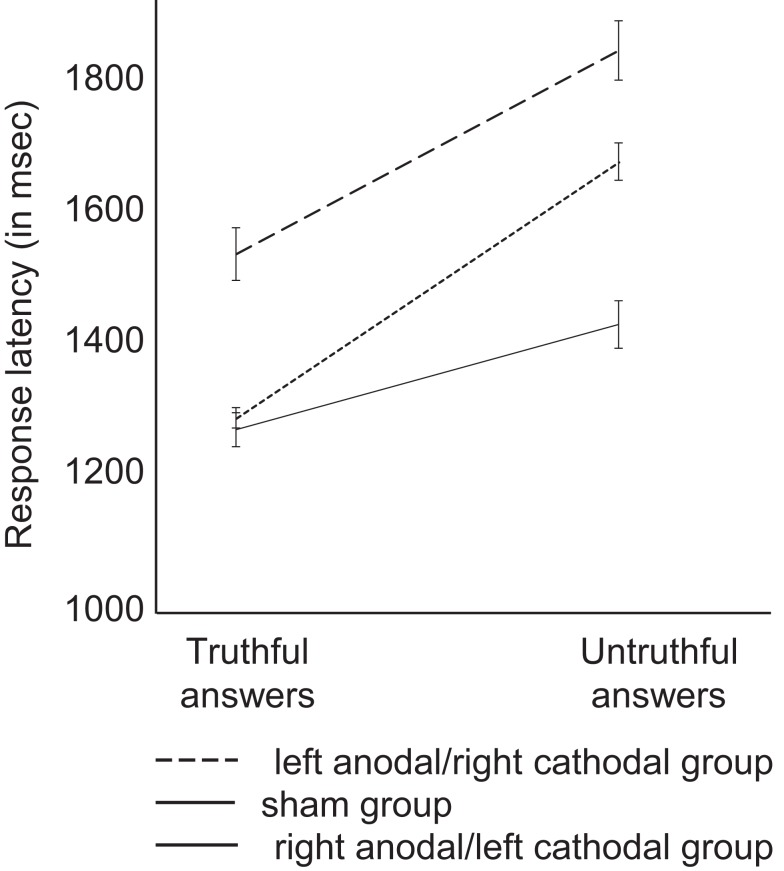
**Averaged response latency of truthful and untruthful answers for each group of participants in Task 1**. Participants who received right anodal/left cathodal DLPFC stimulation showed smaller difference in response latency between truthful and untruthful answers (difference of 185 ms) than the left anodal/right cathodal (difference of 358 ms) and sham groups (difference of 449 ms). Error bars represent SEM.

In Task 1, there was a main effect of stimulation group on response latency [untruthful versus truthful answers; ANOVA; *F*_(2,30)_ = 3.68; *p* = 0.037]. This is illustrated in Figure 2. Bonferroni *post hoc* analysis revealed a difference between the right anodal/left cathodal and sham groups (*p* = 0.036): participants who received right anodal/left cathodal stimulation showed smaller difference in response latency between untruthful and truthful answers as compared to participants who received sham stimulation. There was no significant latency difference between the left anodal/right cathodal and sham groups (*p* = 0.27) and no difference between the two active groups (*p* > 0.1). Also, there was no significant latency difference between women and men (*p* > 0.1). For accuracy (i.e., the number of correct pairs of answers), results revealed no significant group difference across groups (78% of correct pairs for the right anodal/left cathodal group; 85% correct pairs for the left anodal/right cathodal group; and 84% correct pairs for the sham group; *p* > 0.1).

### Experimental Task 2: Deceiving about personal past experience

#### Generating untruthful responses about personal past experience with memorized lies

**Figure 3:**
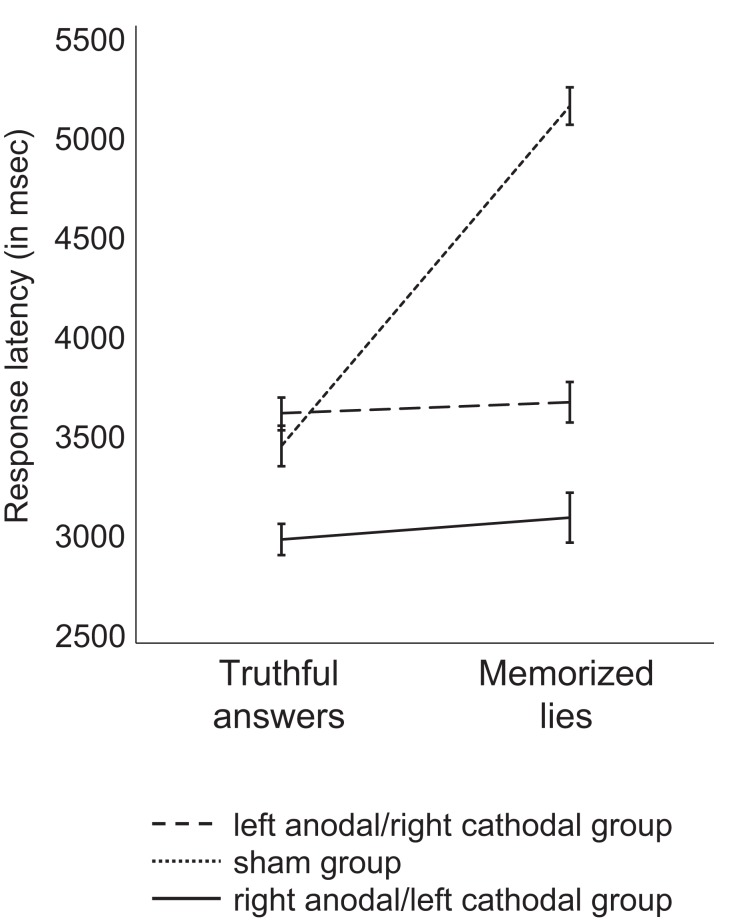
**Averaged response latency of truthful answers and memorized lies for each group of participants in Task 2**. Participants who received active DLPFC stimulation were faster at retrieving memorized lies as illustrated by smaller difference in response latency between truthful and memorized lies (right anodal/left cathodal DLPFC stimulation: difference of 10 ms; left anodal/right cathodal DLPFC stimulation: difference of 6 ms) than the sham groups (difference of 166 ms). Error bars represent SEM.

Results of deception with *memorized lies* revealed an effect of stimulation group on response latency [ANOVA; *F*_(2,35)_ = 4.593; *p* = 0.017]. Bonferroni *post hoc* analysis revealed a significant difference between the sham and the left anodal/right cathodal groups (*p* = 0.035), as well as between the sham and the right anodal/left cathodal groups (*p* = 0.044). As illustrated in Figure 3, response latency difference between memorized untruthful and truthful responses was significantly smaller in subjects who received active stimulation as compared to those who received sham stimulation. There was no difference in response latency between women and men (*p* > 0.1). For accuracy, there was no effect of stimulation group (*p* = 0.095) and no effect of condition (*p* > 0.1). Participants with left anodal/right cathodal, right anodal/left cathodal, and sham stimulation provided correct truthful answers at 92% (SEM = 0.7), 88% (SEM = 1.1), and 82% (SEM = 1.2), respectively, and correct memorized lies at 94% (SEM = 0.8), 91% (SEM = 1.0), and 91% (SEM = 1.1), respectively.

#### Generating untruthful responses about personal past experience with spontaneous lies

**Figure 4:**
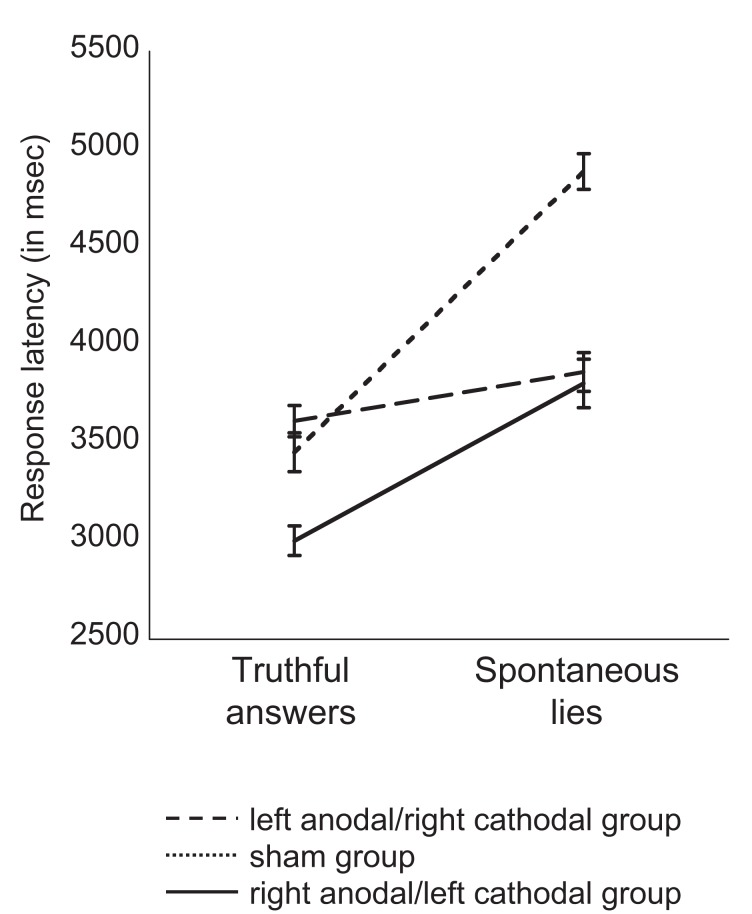
**Averaged response latency of truthful answers and memorized lies for each group of participants in Task 2**. Participants who received left anodal/rightt cathodal DLPFC stimulation were faster at generating spontaneous lies, showing smaller difference between truthful answers and spontaneous lies (difference of 25 ms) than participants who received sham stimulation (difference of 143 ms) and right anodal/left cathodal DLPFC stimulation (difference of 80 ms). Error bars represent SEM.

Results of generating untruthful responses *with spontaneous lies* revealed an effect of stimulation group in response latency [ANOVA; *F*_(2,35)_ = 3.530; *p* = 0.041]. Bonferroni *post hoc* analysis revealed a significant difference in response latency between the sham group and the left anodal/right cathodal stimulation group (*p* = 0.036), but no significant difference between the right anodal/left cathodal and sham groups (*p* > 0.1). As shown in Figure 4, response latency difference between spontaneous untruthful and truthful responses was smaller in subjects who received active stimulation as compared to that who received sham stimulation. There was no difference in response latency between women and men (*p* > 0.1). For accuracy (i.e., the number of coherent untruthful answers), results revealed no effect of stimulation group (*p* = 0.094) and no effect of condition (*p* > = 0.1). Participants with left anodal/right cathodal, right anodal/left cathodal, and sham stimulation provided correct truthful answers at 92% (SEM = 0.7), 88% (SEM = 1.1), and 82% (SEM = 1.2), respectively, and coherent spontaneous lies at 75% (SEM = 1.3), 80% (SEM = 2.0), and 80% (SEM = 1.9), respectively.

### Control Task 3: Generation of spontaneous verbal responses

For the control verbal task, there was a no group effect on response latency [ANOVA; *F*_(2,35)_ = 2.850; *p* = 0.072]. For the number of words, groups did not significantly differed [ANOVA; *F*_(2,35)_ = 0.40; *p* > 0.1]. Volunteers receiving right anodal/left cathodal stimulation produced an average of 7.2 words (SEM = 0.5), those with left anodal/right cathodal stimulation produced an average of 9.5 words (SEM = 0.6), and those with sham stimulation an average of 6.5 words (SEM = 0.3).

### Control Task 4: Stroop interference

**Figure 5:**
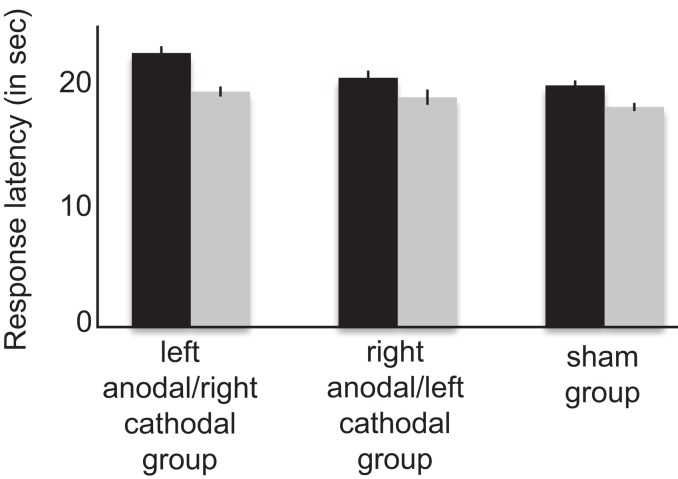
**Averaged response latency for each group of participants at the Stroop interference task**. Participants were faster when they performed the task the second time as compared as the first time, regardless of their stimulation condition. Error bars represents SEM.

For the Stroop task, response latencies were submitted to a repeated measures ANOVA with time of assessment (pre-tDCS, post-tDCS) as within-subjects factor and stimulation groups (right anodal/left cathodal stimulation group, left anodal/right cathodal stimulation group, and sham group) as between subject factor. There was an effect of time of assessment [*F*_(1,11)_ = 16.973; *p* < 0.002], no effect of stimulation group [*F*_(2,22)_ = 0.139; *p* > 0.1], and no interaction between time and group [*F*_(2,22)_ = 0.484; *p* > 0.1] was observed. The effect of time of assessment reflects faster color naming on the second assessment in all three groups of subjects, likely due to repeated testing (Figure 5).

### Psychopathic personality inventory

For the personality profile assessed in participants prior stimulation, there was no group difference for the total PPI score [ANOVA; *F*_(2,61)_ = 0.298; *p* > 0.1] and all the subscales (*p* > 0.1), except for the coldheartedness (*p* = 0.017) and stress immunity subscales (*p* = 0.001). Bonferroni *post hoc* analysis revealed for the coldheartedness subscale a difference between the right anodal/left cathodal and left anodal/right cathodal groups (*p* = 0.014), but no difference between the right anodal/left cathodal and sham groups (*p* > 0.1), or between the left anodal/right cathodal and sham groups (*p* > 0.1). For the stress immunity subscale, there was a difference between the right anodal/left cathodal and left anodal/right cathodal groups (*p* = 0.001) and between the left anodal/right cathodal and sham groups (*p* = 0.029), but no difference between right anodal/left cathodal and sham groups (*p* > 0.1). Scores are presented in Table 1.

## Discussion

Results from this work revealed that non-invasive brain stimulation with tDCS over the DLPFC can modulate production of untruthful answers about subject’s personal life. We observed a reduced response latency associated with untruthful answers, one of the most reliable cues for identifying lies. Our results extend findings from prior brain stimulation studies. In Priori et al. (2008), tDCS over the right DLPFC coupled with anodal/cathodal reduced response latency when subjects had to report through a motor response that they had not seen a picture when they had been previously presented with the picture. In Karim et al. (2009), response latency was shorter in subjects who receive tDCS over the anterior prefrontal cortex when they had to lie at the Guilty Knowledge Test. In Mameli et al. (2010), healthy subjects receiving active anodal tDCS over the both DLPFC cortices were faster at providing lies on general knowledge as compared to that before stimulation. This effect was not observed on lies involving personal information. From an evolutionary point of view, our results and the prior findings support the idea that deception is a relatively new cognitive and neural development (e.g., Premack, 2007), that is a learned behavior that can be influenced via the DLPFC, a highly plastic brain region.

A further novel finding from our work is that the hemispheric contribution was different according to contexts. Right anodal/left cathodal DLPFC stimulation resulted in improvement for generating untruthful answers of relatively guilt-free personal questions on daily activities through motor responses (Task 1) and generating memorized untruthful answers about subjects’ past through verbal responses (Task 2 with memorized lies). The opposite electrode arrangement (left anodal/right cathodal) also improves deceptive skills but only for generating spontaneous and memorized untruthful answers about subjects’ past experience (Task 2). Brain imaging studies contrasting truthful with deceptive answers, found enhanced activity in *bilateral* Brodman Area (BA) 47 in the task we used in Task 1 (Spence et al., 2001), in *bilateral* BA 10 in the task we used in Task 2 with memorized lies, and in *bilateral* BA 10 and right BA 9 in the task we used in Experiment 2B (Ganis et al., 2003). However, our results reveal a laterality to the contributions. In Karton and Bachmann (2011), subjects tended to lie more often were they received repetitive transcranial magnetic stimulation over the left, as compared to the right DLPFC in a task in which subjects were free to lie or not. Neither the present, nor previous brain stimulation studies can conclusively establish whether the impact on deception is solely due to the modulation of activity in one DLPFC, or the result of changing the balance of activity across both DLPFCs as brain activity was not measured. Findings are most cautiously interpreted as a result from modulation of a functionally connected network, with DLPFC as a primary modulated areas, likely including the orbitofrontal area, which has also been involved in deception (Spence et al., 2001, 2004; Ganis et al., 2003). We believe also that it is too soon to speculate on the specific role of each DLPFC in deceptive abilities, but our results suggest a differential contribution according to contexts. Future work should use single electrode arrangement and/or combine non-invasive brain stimulation with neuroimaging to identify the key network involved in deceptive abilities.

There are various cognitive processes required to generate untruthful answers in the present experiments that might have been modulated by tDCS. The cognitive demand required for being deceptive in the present experiments follows to some extent Walczyk et al.’s (2003) model. According to this model, for lies to be produced, cognitive processes control actions in the following way: (1) working memory first activates knowledge of the truth; (2) then decision-making processes are elicited to determine whether or not to lie; (3) then inhibition is required to conceal truthful information; and (4) finally, attention processes mediate knowledge about the context in order to construct a plausible lie. Stages one through three are relevant to cognitive processes involved in Tasks 1 and 2, and stages one through four to that in Task 2 with spontaneous lies. In order to successfully lie here, cognitive processes required included:

(1) Activation of some working memory components (e.g., Task 1: *Did I drink water today?*; Task 2 (with memorized and spontaneous lies): *What was the best show I have seen?*);
(2) Following of the instruction whether or not to generate untruthful answers (task switching);
(3) Suppression of the pre-potent answer to conceal the truth when they had to untruthful answers (e.g., Experiment 1: *Yes I drank water today*; Task 2 with memorized and spontaneous untruthful answers: *The best movie I have seen is Alegria*), and finally;
(4) Generation of the response:
  4(a) Reversal of the answer in Task 1 (e.g., *No*);
  4(b) Retrieval of the memorized lie in Task 2 with memorized untruthful answers (e.g., *Quidam*);
  4(c) Construction of a novel lie in Task 2 with spontaneous untruthful answers (e.g., *Saltimbanco*).

**Table 1:** **Scores for each participant at the Psychopathic Personality Inventory**.

tDCS	ID	Total score	Machiavellian egocentricity	Social potency	Fearlessness	Coldheartedness	Impulsivity non-conformity	Alienation	Carefree non-planfulness	Stress immunity
RA/LC	1	422	65	94	63	39	41	42	41	32
LA/RC	2	302	58	40	32	36	29	38	39	24
SR	3	317	46	52	35	35	33	37	43	30
RA/LC	4	408	75	62	43	48	47	50	48	28
LA/RC	5	331	59	52	40	44	35	27	46	22
SL	6	357	66	62	46	34	34	42	45	22
RA/LC	7	342	51	50	48	45	34	38	45	25
LA/RC	8	401	70	75	47	39	45	47	49	21
SR	9	323	63	51	34	35	35	30	45	22
RA/LC	10	322	56	48	39	49	28	23	41	35
LA/RC	11	351	60	55	49	34	40	36	50	22
SL	12	375	78	56	45	50	41	39	33	27
RA/LC	13	398	63	67	57	49	41	38	44	31
LA/RC	14	385	68	72	55	25	46	49	41	24
SR	15	344	54	72	47	36	38	35	29	26
RA/LC	16	334	50	53	43	39	39	35	39	30
LA/RC	17	311	52	53	46	37	38	24	35	20
SL	18	349	73	47	45	35	29	42	39	30
RA/LC	19	340	75	58	30	39	32	37	39	25
LA/RC	20	380	67	76	46	28	39	48	48	19
SR	21	323	54	60	34	35	43	25	43	25
RA/LC	22	291	53	39	38	26	35	39	42	16
LA/RC	23	383	70	64	45	44	41	45	37	30
SL	24	343	66	53	45	39	40	29	38	23
RA/LC	25	312	55	57	32	33	29	41	34	26
LA/RC	26	382	72	56	40	35	50	54	48	20
SR	27	360	67	69	48	35	32	39	34	29
RA/LC	28	310	66	37	31	38	31	43	31	27
LA/RC	29	309	58	47	38	35	36	39	29	22
SL	30	333	48	50	53	35	40	35	38	29
RA/LC	31	377	61	53	64	41	43	34	46	30
LA/RC	32	351	65	65	40	37	40	40	33	28
SR	33	365	62	57	52	47	45	29	38	30
RA/LC	34	335	58	63	42	39	32	34	36	24
LA/RC	35	359	63	50	54	36	46	43	37	23
SL	36	367	57	60	56	37	45	41	38	27

We discuss some potential cognitive functions that might have been impacted in a different way across DLPFC stimulation conditions according to the observed improved production of untruthful responses skills. First, one could argue that DLPFC modulation might have differentially impacted working memory load across stimulation groups. We believe this is unlikely the case. Memory has to be activated for both truthful and untruthful answers between groups. Therefore, if neuromodulation had impacted memory, there would have been a difference in latency of truthful answers, which was not observed in any of our experiments. In addition, if neuromodulation had significantly affected memory access, there would likely be a difference in the number of incorrect answers between groups. This again, was not observed.

Second, one could argue that DLPFC modulation reduced the demands of cue-elicited behavior as subjects were instructed for each trial to be untruthful or truthful as task switching can elicit activation in the prefrontal cortex (e.g., Dove et al., 2000). However, this demand was required in both deceptive and truthful conditions and subjects were not faster at providing truthful answers.

Third, an important cognitive process required for providing untruthful answers in our three experiments was to refrain from emitting relatively pre-potent responses. However inhibition was not facilitated with active tDCS over the DLPFC at the Stroop interference paradigm. If neuromodulation had changed inhibition, this facilitation would have been selective for deceptive behaviors. This would suggest that inhibitory systems are fundamentally different between inhibiting a truthful answer (even for benign white little lies as those in Task 1) and suppressing naming color incongruent words at the Stroop task.

Fourth, volunteers in Task 1 had to provide the deceptive answer by reversing the pre-potent response. Although the answer was a simple *yes* or *no* motor response, we cannot rule out that this reversal process might have been improved by right anodal/left cathodal stimulation. However, in Task 2 with memorized untruthful answers there was no such reversal of the pre-potent response and subjects were still with this same electrode arrangement (right anodal/left cathodal stimulation).

Fifth, deceptive abilities are entangled with other cognitive processes such as producing coherent and novel verbal responses, a process that was required to successfully generate untruthful answers in Task 2 with spontaneous lies. One could argue that left anodal/right cathodal stimulation might have speed up the process of generating novel information. However this was not observed in subjects who received this stimulation condition in the control experiment (*Generation of spontaneous verbal responses*). In line with this, emotional state can also be involved during deception. We cannot rule out the impact of the observed group difference of coldheartedness and stress level on untruthful answers. However, in the case of coldheartedness, the difference was between the two active groups. Thus, the improved deceptive responses between the right anodal/left cathodal stimulation and sham groups observed in Tasks 1 and 2 was unlikely due to the coldhertedness scores. For the stress immunity subscale, the difference was observed between the two active groups as well as between the left anodal/right cathodal and the sham groups. We cannot rule out that this latter difference could have played a role in the improved deceptive abilities observed in Task 2 with spontaneous untruthful answers. No group difference was however found for the total PPI score or the other subscales: Machiavellian egocentricity, social potency, fearlessness, coldheartedness, impulsive non-conformity, blame externalization, and carefree non-planfulness.

It is possible that DLPFC modulation might have reduced the overall cognitive effort usually associated with deceptive behaviors (Vrij and Mann, 2001). This effect would have been selective for generating untruthful answers as no improved performance was observed in the truthful condition, in the Stroop interference paradigm, or in the control verbal response experiment. Future experiments are warranted to measure changes in cognitive effort with brain stimulation to test whether or not it is specific to deceptive abilities. Other processes should be tested in future studies such as the act of deliberation over deception, weighting of risk and benefits, the mind of the other(s) to be lied to, and the content of the lie. Also, we studied generation of untruthful responses using cue-elicited tasks. Future studies should test shared and differential cognitive processes involved between external (as in these tasks) and internal cued lies (as done in Karim et al., 2009; Karton and Bachmann, 2011).

Of particular note, a lengthened response latency has been suggested as one the most reliable indicators of deceit to classify liars from truth-tellers (Vrij et al., 2008; see also Spence et al., 2001, 2004; DePaulo et al., 2003; Farrow et al., 2003; Nunez et al., 2005) and difficult to explicitly manipulate (Seymour et al., 2000). Here, although the length difference between being untruthful and truthful was significantly diminished after volunteers received active stimulation as compared to those who received sham stimulation, making them *better (faster)* liars, they still remained slightly slower when providing untruthful as compared to truthful answers.

We want to stress justifiable ethical and legal concerns raised by Canli et al. (2007) and Luber et al. (2009) in future work. We believe the risk-benefit ratio of understanding the neurobiological and cognitive foundation of deception and how it can be modulated is justified because it might by possible to develop protocols leading to clinical benefits in various clinical populations in which processing deception is a major disability, such as in patients with antisocial personality disorder, Parkinson’s disease, or with frontal lesions. However, studies have to be conducted under proper oversight and investigators have to be aware of the potential implications of their work.

## Conflict of Interest Statement

The authors declare that the research was conducted in the absence of any commercial or financial relationships that could be construed as a potential conflict of interest.
